# Macro- and Micro-Expressions Facial Datasets: A Survey

**DOI:** 10.3390/s22041524

**Published:** 2022-02-16

**Authors:** Hajer Guerdelli, Claudio Ferrari, Walid Barhoumi, Haythem Ghazouani, Stefano Berretti

**Affiliations:** 1 Research Team on Intelligent Systems in Imaging and Artificial Vision (SIIVA), LR16ES06 Laboratoire de Recherche en Informatique, Modélisation et Traitement de’Information et dea Connaissance (LIMTIC), Institut Supérieur d’Informatique d’El Manar, Université de Tunis El Manar, Tunis 1068, Tunisia; hajer.guerdelli@unifi.it (H.G.); walid.barhoumi@enicarthage.rnu.tn (W.B.); haythem.ghazouani@enicarthage.rnu.tn (H.G.); 2 Media Integration and Communication Center, University of Florence, 50121 Firenze, Italy; 3 Department of Engineering and Architecture, University of Parma, 43121 Parma, Italy; claudio.ferrari2@unipr.it; 4 Ecole Nationale d’Ingénieurs de Carthage, Université de Carthage, Carthage 1054, Tunisia

**Keywords:** macro-expressions datasets, micro-expressions datasets, facial expression recognition, applications of facial expression datasets

## Abstract

Automatic facial expression recognition is essential for many potential applications. Thus, having a clear overview on existing datasets that have been investigated within the framework of face expression recognition is of paramount importance in designing and evaluating effective solutions, notably for neural networks-based training. In this survey, we provide a review of more than eighty facial expression datasets, while taking into account both macro- and micro-expressions. The proposed study is mostly focused on spontaneous and in-the-wild datasets, given the common trend in the research is that of considering contexts where expressions are shown in a spontaneous way and in a real context. We have also provided instances of potential applications of the investigated datasets, while putting into evidence their pros and cons. The proposed survey can help researchers to have a better understanding of the characteristics of the existing datasets, thus facilitating the choice of the data that best suits the particular context of their application.

## 1. Introduction

In recent years, we assisted to a remarkable proliferation of facial-expression datasets. One main reason for this is the advancement in the facial expression recognition (FER) research, which is primarily motivated by the interest in the many potential applications it may have (e.g., in the medical domain to detect signs of depression or pain, in smart driving to interact with future vehicles endowed with increasing intelligence, in social marketing applications, and in human computer interaction, to cite a few). Though facial expressions are innate in humans expressiveness, their relation to emotions isess delineated, so it is first relevant to explore, in some detail, the relation and differences between facial expressions and human emotions. An emotion is a complex and intense psycho-physiological experience of an individual’s state of mind when reacting to biochemical (internal) and environmental influences (external) [1]. For humans, emotions fundamentally include “physiological behavior, expressive behaviors and consciousness” [2]. Paul Ekman theorized that some basic human emotions are innate and shared by everyone, and that they are accompanied across cultures by universal facial expressions. Therefore, according to Ekman, an emotion causes facial expressions. However, some researchers have found that reproducing the facial expressions causes the corresponding emotion [3]. By making a happy face, for example, we begin to feel happiness. Therefore, facial expressions also cause emotions. This suggests to us that emotions and facial expressions are strongly correlated. However, according to the study of [4], the face does not always tell us the truth about emotions. At one extreme, the work of [5] has found no clearink between facial movements and internal emotional states. It is worth mentioning that almost all the studied datasets are based on the assumption that the same emotion causes nearly the same facial expressions. Indeed, within the framework of posed expressions the subject is asked to express him/herself for a given emotion. Differently, for in-the-wild applications and spontaneous expressions, the ground truths are made based on the opinions of experts who assign an emotion to the subject’s face according to his/her facial expressions [6]. We can, therefore, state that FER datasets are benchmarks for the facial expression classification rather than the emotion recognition.

The state-of-the-art methods have progressed up to aevel that has made them perfectly accurate for the first datasets, which were of reduced size while being acquired in *posed* conditions. This performance saturation on standard benchmarks [7] has induced the collection of new and more challenging datasets. In this respect, one trend is represented by the shift from *posed* to *spontaneous* and *in-the-wild* capturing conditions. In particular:

- *Posed* datasets are typically acquired by asking the subjects to show one of the six basic expressions as defined by Ekman [8]. In most of the cases, experienced actors are enrolled, and capturing takes place in constrainedaboratory conditions;
- *Spontaneous* datasets include expressions that are stimulated by the participants. For instance, this can be the result of watching a video or of a face-to-face interaction. Participants are aware that they are monitored, but emotions are shown in a natural way, rather than acted. In most of the cases, the acquisition context is a constrained one;
- *In-the-wild* datasets relax any acquisition constraint, and expressive subjects are filmed in real-world scenarios. This is obtained by analyzing facial expressions in images and videos in movies, talk-shows, interviews, etc.

Furthermore, newly proposed facial expression datasets try to fill the gap between data and algorithms. They provide the amount of variegated data that allow facial expression recognition solutions based on neural networks toearn effective internal weights. In the recently introduced datasets, the effort of providing more annotations than those given in the oldest ones is also evidently noticed. These annotations include more expressions than the six ones categorized in the Ekman’s model, and also some emotional states. For instance, additional states have been included as follows: the neutral state in the iSAFE [9], AFEW [10], and FER-2013 [11] datasets, the neutral state and the pain state in Hi4D-ADSIP [12], the neutral state and the contempt one in BAUM-2 [13], and the embarrassment and the pain emotions in BP4D-Spontaneous [14]. Moreover, emotions have been categorized into nine categories in FER-Wild [15], thirteen emotional and mental states in BAUM-1 [16], and twenty-three categories of emotion in EmotioNet [17].

**Figure 1:**
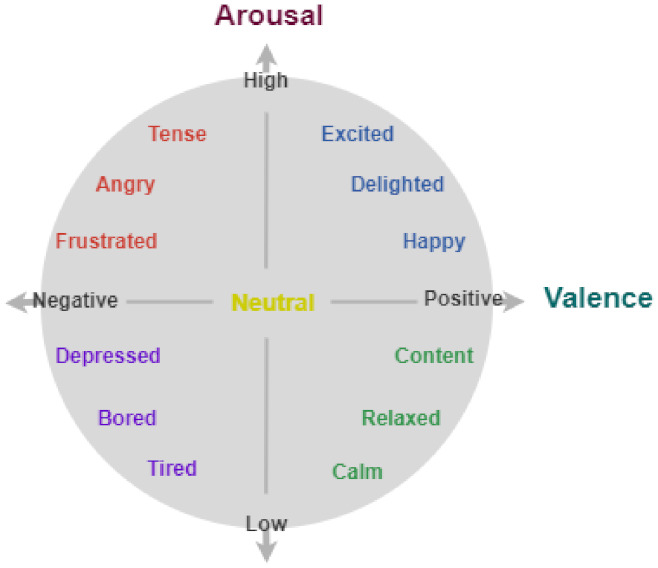
The valence–arousal continuous emotional space.

In addition, there are also datasets that propose more continuous models based on the *valence* of the expression (either positive or negative) and its intensity (called *arousal*). According to this model, as proposed by Russel [18], expressions are regarded as continuously distributed in a 2D chart, where the horizontal axis isabeled with valence values from *displeasure* to *pleasure*, while arousal passes are sorted fromow- to high-activation along the vertical axis (the center of the circle represents a neutral valence and a mediumevel of arousal [19]). Using this diagram, several combinations are possible (see Figure 1), according to the different quadrants:

- *First quadrant*—emotional states go from pleased (high valence, medium arousal) to excited (about neutral valence, high arousal);
- *Second quadrant*—high arousal with about neutral valence here indicates an alarmed state, while high-negative valence and medium arousal bring to a frustrated state;
- *Third quadrant*—in this quadrant, high-negative valence and medium arousal indicate sad/depressed condition, while the status withow arousal and about neutral valence corresponds to a tired state;
- *Fourth quadrant*—finally, in this quadrant forow arousal and about neutral valence a calm/sleepy state is valence and medium arousal.

The states reported above are just given to exemplify the representations in the continuous space obtained by moving on the boundary circle of the 2D chart, while many other emotional states can be defined by moving inside the circle.

In the above overview, we have implicitly referred to *macro-expression* datasets that are normally indicated just as facial expression datasets, while omitting the “macro-” prefix. Actually, research on facial expressions can be also conducted by analyzing *micro-expressions*. These are unconscious reactions to emotional states thatast only a fraction of the time of macro-expressions (less than half a second). This poses a series of additional challenges. Though micro-expressions can be categorized in the same way as the macro ones, they are subtle and difficult to observe with a normal camera working at 25 frames per second. Furthermore, since they are unconscious reactions, micro-expressions have proven to be difficult to hide and also to act. This makes capturing data for micro-expression datasets, as well as annotating them in a reliable way, quite complicated. In particular, this would require the adoption of cameras with high frame rates (up to 100/200 frames per second). It is also worth mentioning that spontaneous and in-the-wild emotions induced by specific video clips are very challenging toabel. There are mainly two procedures used toabel uncontrolled emotions. For the first one, as used toabel the DISFA and the MMI datasets, the data are annotated based on the Facial Action Coding System (FACS), which is a coding of facial muscle actions. The second procedure uses self-reported data of subjects as the real emotionabels as performed, for example, toabel the USTC-NVIE dataset. Therefore, many challenges are intrinsic to the process of facial expression datasetabeling. First, different emotions can act on the same facial muscles, such as glaring and raising the chin, which are often spotted for both disgust and fear emotions. Second, unlike posed expressions, spontaneous emotions display may vary from one person to another, which makes their annotation more difficult. Third, relying on self-reported data makes the dataset annotation subjective. Finally, the absence of a protocol to unify these procedures can be an obstacle to conduct deeper investigations to determine their influence on emotion detection.

All the above considered, we provide in this survey an overview of the existing datasets for facial expression analysis by categorizing them as oriented to *macro-* and *micro-expression* recognition. Actually, most of the existing datasets fall into the first category, mainly because macro-expressions are easier to collect and detect than micro-expressions. In particular, we are not aware of works that have summarized, in a systematic way, the existing datasets for both macro- and micro-expression recognition. Due to the increasing number of datasets, and their different characteristics, we believe that this review can provide researchers with a useful guide for orienting the choice of the adequate datasets for training and testing their models. In fact, for both macro- and micro-expressions dataset, we have identified several features that characterize and make specific each dataset. The main distinction we used is between *spontaneous* and *posed* datasets, on the one hand, and datasets acquired *in-the-wild*, on the other hand. In fact, these result in different acquisition requirements, protocols andabeling that ultimately open the way for investigating different challenges.

Thus, in this survey, we will follow the above categorization in presenting the existing datasets, but we do not refer to posed datasets. The reason for this is that most of the posed datasets were collected in the early stage of the research on macro-expression recognition. Therefore, compared to the benchmarks used in the currentiterature, such datasets have a small size with the saturated performance shown by the state-of-the-art methods. We chose to divide the proposed survey into two main sections, i.e., one for macro- and one for micro-expressions datasets, each of which is divided in two subsections, i.e., for spontaneous and in-the-wild data, respectively. We enclose eighty datasets, covering both publicly available and not publicly available ones in order to provide a comprehensive overview. We described each dataset, and categorized it based on particular characteristics such as number of subjects, age, frame per second, ethnicity and amount of data. Table 1 summarizes the proposed classification of macro- and-micro-expression datasets. Unlike other facial expression datasets surveys, such as that of Khan et al. [20], where twenty-seven datasets were divided into video-based and image-based, our survey takes into account several different and general aspects, and encloses eighty datasets. For instance, in [21], authors have structured their survey according to two session datasets and face emotion recognition methods and technologies, where only eleven datasets have been discussed.

The remaining of this paper is organized as follows. In Section 2, we introduce the main characteristics that define the content of a macro-expression dataset, before summarizing the content of 70 existing datasets. In Section 3, we provide the same analysis for the case of micro-expression datasets. Some applications that used the macro- and micro-expression datasets are given in Section 4. Finally, we discuss and conclude the paper in Section 5.

## 2. Macro-Expression Datasets

A macro-expression dataset is intended as a collection of images or videos of subjects that exhibit a facial expression as a consequence of an emotional state. There are also collections of static and dynamic three-dimensional (3D) scans of the face that capture the same range of emotions as for the 2D counterparts. A common trend that can be observed is these datasets is that of capturing facial expressions that go one step further than the strict categorization provided by the Ekman’s theory [22,23]. In fact, while Ekman proposed the expression categorization into six universal categories (i.e., angry, disgust, fear, happy, sad and surprise) there is now the convincement that, despite this basic categorization being useful for a high-level view, it is too schematic to span the broad spectrum of human facial expressions. Therefore, other insights have made their way, with the *circumplex* model [18] being one of the most impactful. In addition to the type of the collected data (either images, videos or 3D scans), the capturing conditions and the expression model, the existing macro-expression datasets can be characterized according to several other features. In particular, we have identified the following features:

- *Number of subjects*: The existing datasets vary between four and thousands of subjects. The number of different individuals is particularly relevant for methods that needarge quantities of data toearn models capable of generalizing to unseen identities;
- *Age*: Enrolled subjects vary from infants to young children and elderly people;
- *Frames per second (FPS)*: This can vary depending on the application context. For instance, to study the facial expression dynamics, a high FPS can help, whereasow FPS is often adopted for samples captured in real-life conditions;
- *Ethnicity*: Variability in terms of ethnic groups such as Caucasian, Latino, Asian, Black or African American, East-Asian, South-Asian, Turkish, etc., can be relevant and is typically a desired feature in collecting expression datasets;
- *Amount of data*: Number of images, videos or video frames.

Furthermore, datasets are usually accompanied with annotations that are essential for training, testing and validating methods for facial expression recognition. These annotations are particularly relevant for videos where, depending on the fact the annotations are given at frame or videoevel, analysis at different granularity can be performed. This has a considerable impact depending on whether the datasets include posed, spontaneous or in-the-wild capturing, and on the expression model, either based on the six basic expressions or the circumplex model. In fact, while providing the six expressionabels for posed and spontaneous datasets is an easy task, some more difficulties are experienced when the circumplex model is adopted. For in-the-wild capturing, ground-truth annotations are provided offline, and require experienced annotators. This introduces aot of work from human annotators, which is costly and time-consuming. Sometimes, this human effort is alleviated by resorting to some form of *Mechanical Turk* that distributes theoad to low-experienced andow-cost workers. However, being performed by non-expert personnel, the resulting annotations can show a diminished accuracy being originated by averaging annotations across several mechanical workers.

### 2.1. Spontaneous Datasets

**Figure 2:**
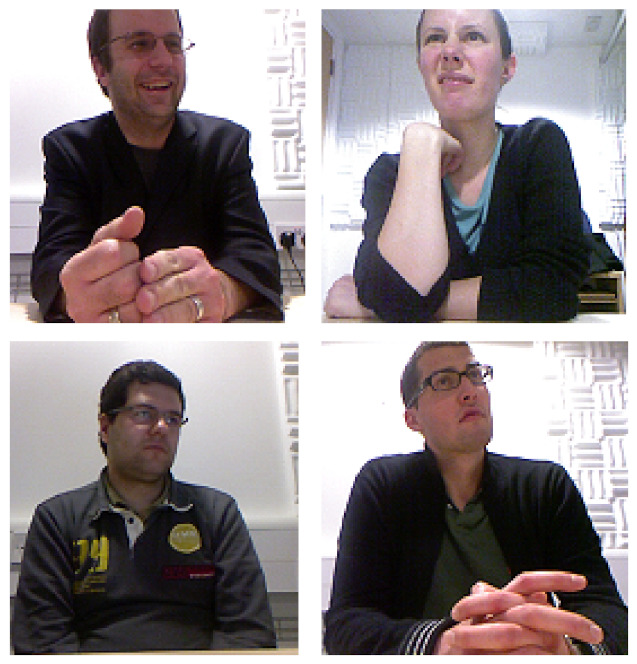
Sample frames from the CAM3D spontaneous dataset.

In this section, we focus on spontaneous macro-expression datasets. Some samples of these expressions are shown in Figure 2. These datasets areisted in Section 3.4.

**EB+ (An expanded version of BP4D+)**: The EB+ [24] dataset is an expanded version of BP4D+ [25]. It contains videos from a total of 200 subjects: 140 subjects from BP4D+, plus 60 additional subjects associated with five to eight tasks that involve inductions of varied emotions of a participant interacting with an experimenter. The emotions are inducted when the participants interact with the experimenter. A certified FACS coders team annotated the dataset manually.

**Figure 3:**
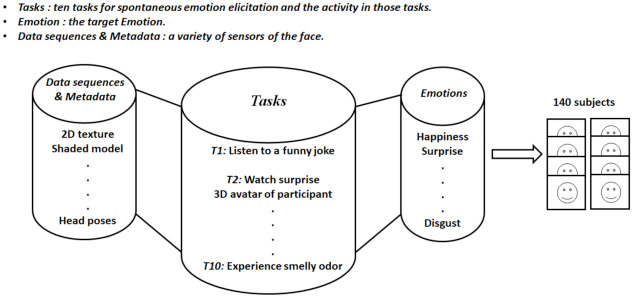
Structure of the BP4D+ dataset.

**BP4D+ (Multimodal Spontaneous Emotion)**: Those tasks in EB+ are minutely explained in the BP4D+ or MultiModal Spontaneous Emotion (MMSE) dataset. This dataset is collected for human behavior analysis, and it illustrates 140 participants from different ethnic origins. The collected data included thermal (infrared) sensing, high-resolution 2D videos, high-resolution 3D dynamic imaging and contact physiological sensors that included respiration, heart rate, electrical conductivity of the skin and blood pressure. BP4D+ (see Figure 3) presents ten different emotion categories (happiness or amusement, surprise, sadness, startle or surprise, skeptical, embarrassment, fear or nervous, physical pain, angry and disgust) recorded per person according to the ten tasks that each person experienced. More specifically, these tasks include:isten to a funny joke, watch 3D avatar of participants, listen to 911 emergency phone calls, experience a sudden burst of sound, response to true or false question, improvise a silly song, dart game, submerge hands into ice water, complained for a poor performance and smell a smelly odor. BP4D+ has aarger scale and variability for images than BP4D Spontaneous [14]. Since its creation, BP4D+ has being widely used.

**BP4D (Binghamton-Pittsburgh 3D DynAMIc Spontaneous Facial Expression Data-base)**: BP4D Spontaneous [14] contains 41 participants from four different ethnic origins (Asian, African-American, Hispanic, and Euro-American). It presents eight emotions (happiness or amusement, sadness, surprise or startle, embarrassment, fear or nervous, pain, anger or upset and disgust) derived through a combination of interviews, planned activities, film watching, cold pressor test, social challenge and olfactory stimulation. The facial expressions in the dataset had been annotated using the Facial Action Coding System (FACS).

**iSAFE (Indian Semi-Acted Facial Expression Database)**: iSAFE [9] contains 44 volunteers from Indo-Aryan and Dravidian (Asian), 395 clips and seven emotions (happy, sad, fear, surprise, angry, neutral, disgust) captured with a camera behind aaptop, where the volunteers were asked to watch a few stimulant videos. The facial expressions were manually self-annotated by a user-interface portal and cross annotated by an annotator.

**TAVER (Tri-modal Arousal-Valence Emotion Recognition database)**: TAVER [26] contains 17 subjects from one ethnic origin (Korean). It presents a novel method that estimates dimensional emotion states taking color, depth, and thermal recording videos through human–human interaction. The emotion (arousal–valence) was elicited through embarrassing and stressing people by asking them questions in a differentanguage (English) than their own (Korean). The participants self-report feeling uncomfortable for the interviews with anotheranguage. Six human operators annotated the video sequence, with three annotators for each video sequence for more accuracy.

**RAVDESS (Ryerson Audio-Visual Database of Emotional Speech and Song)**: The RAVDESS [27] dataset contains 24 participants from different ethnic groups (Caucasian, East-Asian, and Mixed (East-Asian Caucasian, and Black-Canadian First nations Caucasian)). The emotional elicitation in RAVDESS dataset is done through the true performance of emotion by actors. Actors were told to induce the desired state and provide genuine expressions of emotion. This dataset is particularly suited to machineearning approaches involving supervisedearning.

**GFT (Group Formation Task)**: GFT [28] contains 96 participants and 172,800 frames from aarger study on the impact of alcohol on group formation processes. In this study, participants affirmed that they could comfortably drink ateast three drinks in 30 min. They were seated around a circular table in an observation room where they were asked to consume a beverage and to discuss any topics except theirevel of intoxication.

**SEWA (Automatic Sentiment Analysis in the wild)**: SEWA [29] contains 398 participants of different nationality (British, German, Hungarian, Greek, Serbian, and Chinese), and 1990 audio-visual recording clips were collected during the experiment, comprised of 1600 min of audio-visual data of people’s reaction to adverts and 1057 min of video-chat recordings. To stimulate the emotions, the participants were asked to watch four advertisements, each being around 60 song. These adverts had been chosen to elicit mental states including amusement, empathy, liking and boredom. In a second part, the participants were divided into pairs based on their cultural background, age and gender (for natural interaction, each pair was required to know each other personally in advance). After watching the fourth advertisement, the two participants were asked to discuss, for three minutes on average, the advertisement they had just watched. The subtle changes in the participant’s emotional state (valence, arousal, andiking/disliking) were annotated by human operators from the same cultural background of the recorded subjects (five for each). The SEWA dataset contains annotations for facialandmarks, acousticow-level descriptors, hand gestures, head gestures, facial action units, verbal and vocal cues, continuously valued valence, arousal andiking/disliking, template behaviors, episodes of agreement/disagreement and mimicry episodes.

**BioVid Emo (psychophysiological signals with video signals for discrete basic emotions)**: The BioVid Emo [30] dataset combines psycho-physiological signals with video signals for discrete basic emotions that were effectively elicited by film clips from 86 participants. The psycho-physiological signals that have been considered in this study are: skin conductanceevel, electrocardiogram, trapezius electromyogram and four video signals. Five discrete emotions (amusement, sadness, anger, disgust and fear) were elicited by 15 standardized film clips.

**ISED (Indian Spontaneous Expression Database)**: ISED [31] contains 50 Indian subjects and 428 videos. Emotions were induced among the participants by using emotional videos and simultaneously their self-ratings were collected for each experienced emotion (sadness, surprise, happiness, and disgust).

**4D CCDb (4D Cardiff Conversation Database)**: 4D CCDb [32] contains four participants recording 17 conversations, which have been fully annotated for a speaker andistener activity: conversational facial expressions, head motion, and verbal/non-verbal utterances. The annotation tracks included were: front channel, backchannel, agree, disagree, utterance (verbal/non-verbal), happy (smile oraugh), interesting-backchannel, surprise-positive, surprise-negative, thinking, confusion, head nodding, head shake, head tilt and other.

**MAHNOB Mimicry (The mahnob mimicry database: A database of naturalistic human interactions)**: MAHNOB Mimicry [33] contains 60 subjects from staff and students at Imperial College London (Europe or the Near-East). The subjects were recorded over 54 sessions of dyadic interactions between 12 confederates and their 48 counterparts, being engaged either in a socio-political discussion or negotiating a tenancy agreement.

**OPEN-EmoRec-II (A Multimodal Corpus of Human-Computer Interaction)**: OPEN-EmoRec-II [34] has been designed in order to induce emotional responses in HCI users during two different parts of a HCI-experiment. It contains 30 subjects involving video, audio, physiology (SCL, respiration, BVP, EMG Corrugator supercilii, EMG Zygomaticus Major) and facial reaction annotations.

**AVEC’14 (Audio-Visual Emotion recognition Challenge (AVEC 2014))**: AVEC’14 [35] contains 84 German subjects with 300 audio-visuals. The challenge has two goalsogically organized as sub-challenges: to predict the continuous values of the affective dimensions valence, arousal and dominance at each moment in time; and to predict the value of a single self-reported severity of depression indicator for each recording in the dataset.

**DISFA (A Spontaneous Facial Action Intensity Database)**: DISFA [36] contains 27 subjects from different ethic (Asian, Euro American, Hispanic, and African American) and 130,000 annotations. Participants viewed a four-minute video clip intended to elicit spontaneous Action Units (AUs) in response to videos intended to elicit a range of facial expressions of emotion.

**RECOLA (REmote COLlaborative and Affective interactions = Multimodal Corpus of Remote Collaborative and Affective Interactions (in French: RECOLA))**: RECOLA [37] contains 46 subjects of different nationality (French, Italian, German and Portuguese). It is based on a study focusing on emotion perception during remote collaboration, where participants were asked to perform individual and group tasks.

**AVEC’13 (Audio-Visual Emotion recognition Challenge (AVEC 2013))**: AVEC’13 [38] contains 292 German subjects and 340 audio-visuals. Subjects performed a human-computer interaction task, while being recorded by a webcam and a microphone.

**CCDb (Cardiff conversation database)**: The CCDb [39] 2D audiovisual dataset contains natural conversations between pairs of people. All 16 participants were fully fluent in the Englishanguage. It includes 30 audio-visuals.

**DynEmo (Dynamic and spontaneous emotional facial expression database)**: The DynEmo [40] dataset contains 358 Caucasian participants filmed in natural but standardized conditions. The participants were enrolled into ten tasks to display a subjective affective state rated by both the expresser (self-reported after the emotion inducing tasks, using dimensionally, action readiness and emotionalabels items) as well as the observers (continuous annotations).

**DEAP (A Database for Emotion Analysis Using Physiological Signals)**: DEAP [41] contains 32 mostly European students and 40 videos. Participants watched music videos and rated them on a discrete nine-point scale for valence, arousal and dominance.

**SEMAINE**: SEMAINE [42] contains 24 undergraduate and postgraduate students between 22 and 60 years old. It consists of 130,695 frames of typical session duration for Solid SAL (Sensitive Artificial Listener) and semi-automatic SAL. In these sessions, participants were asked to change character when they got bored, annoyed or felt they had nothing more to say to the character.

**MAHNOB-HCI (multimodal database for affect recognition and implicit tagging)**: MAHNOB-HCI [43] illustrates 27 participants from different educational backgrounds, from undergraduate students to postdoctoral fellows, with different English proficiency from intermediate to native speakers. Participants were shown fragments of movies and pictures, while monitoring them with six video cameras, a head-worn microphone, an eye gaze tracker, as well as physiological sensors measuring ECG, EEG (32 channels), respiration amplitude, and skin temperature.

**UNBC-McMaster (McMaster University and University of Northern British Columbia (UNBC)–Painful data: The UNBC-McMaster shoulder pain expression archive database)**: The UNBC-McMaster (UNBC Shoulder Pain Archive (SP)) [44] dataset contains 25 participants who were self-identified as having a problem with shoulder pain. It contains physical pain/temporal expressions/spontaneous facial expressions relating to genuine pain, while discriminating 48,398 frames/200 video sequences.

**CAM3D (3D corpus of spontaneous complex mental states)**: CAM3D [45] (Figure 2) contains 16 participants from different ethnic backgrounds (Caucasian, Asian and Middle Eastern). It involves 108 videos, where the use of hand-over-face gestures as a novel affects cues for automatic inference of cognitive mental states.

**B3D(AC) (A 3-D Audio-Visual Corpus of Affective Communication)**: The B3D(AC) [46] audio-visual corpus dataset contains 14 participants native English speakers and 1109 sequences. The annotation of the speech signal includes: transcription of the corpus text into the phonological representation, accurate phone segmentation, fundamental frequency extraction, and signal intensity estimation of the speech signals.

**CK+ (Extended Cohn-Kanade Dataset)**: CK+ [47] contains 593 sequences, where the 123 participants have performed series of 23 facial displays. It involves seven emotion categories.

**AvID (Audiovisual speaker identification and emotion detection for secure communications)**: AvID [48] contains 15 subjects, recorded while they describe neutral photographs, play a game of Tetris, describe the game of Tetris and solve cognitive tasks. A one-hour video is captured for each subject, discriminating four class emotions (neutral, relaxed, moderately aroused and highly aroused).

**AVIC (Audiovisual Interest Corpus)**: AVIC [49] contains 21 participants from Asian and European ethnic groups, while involving 324 episodes that consist of spontaneous as well as conversational speech demonstrating “theoretical” effectiveness of the approach.

**DD (Detecting depression from facial actions and vocal prosody)**: The DD dataset [50] illustrates 57 participants from a clinical trial for treatment of depression. Trials were conducted using the Hamilton Rating Scale for Depression (HRS-D), which is a criterion measure for assessing the severity of depression. Participant facial behavior was registered in response to the first three of 17 questions in the HRS-D, such that the questions concerned core features of depression: depressed mood, guilt, and suicidal thoughts.

**SAL (The Sensitive Artificial Listener)**: The SAL [51] dataset is based on the observation that it is possible for two people to have a conversation in which one paysittle or no attention to the meaning of what the other says and chooses responses on the basis of superficial cues. SAL provides a context in which sustained emotionally colored human–machine interaction seems to be achievable. It identifies the four users’ emotional state itself during sessions of 30 min for each user, using evidence from faces, upper body, voice, and key words. The range of emotions is wide, but they are not very intense.

**HUMAINE (The HUMAINE Database: Addressing the Collection and Annotation of Naturalistic and Induced Emotional Data)**: HUMAINE [52] contains 50 clips selected to cover material showing emotion in action and interaction spanning a broad emotional space (positive and negative, active and passive), selected from the following corpora: the Belfast Naturalistic dataset (in English, naturalistic, ten clips), the Castaway Reality Television dataset (in English, naturalistic, ten clips), Sensitive Artificial Listener (in English, induced, 12 clips), Sensitive Artificial Listener (in Hebrew, induced, one clip), Activity/Spaghetti dataset (in English, induced, seven clips), Green Persuasive dataset (in English, induced, four clips), EmoTABOO (in French, induced, two clips), DRIVAWORK corpus (in German, induced, one clip), and GEMEP corpus (in French, acted, one clip).

**EmoTABOO (Collection and Annotation of a Corpus of Human-Human Multimodal Interactions: Emotion and Others Anthropomorphic Characteristics: consisting inetting pairs of people play the game “Taboo”)**: EmoTABOO [53] is a French dataset containing ten audiovisual clips collected during game playing. People were playing at Taboo, a game in which one person has to explain to the other using gestures and body movement a ‘taboo’ concept or word. It involves multimodal interactions between two people and provides an emotional content, with a range of emotions including embarrassment, amusement, etc.

**ENTERFACE**: ENTERFACE [54] includes acquisitions for three multimodal emotion detection modalities: the first modality is given by brain signals via fNIRS and contains 16 participants; the second modality includes face videos of five participants; and the third modality captures the scalp EEG signals of 16 participants. EEG and fNIRS provided an “internal”ook at the emotion generation processes, while video sequences gave an “external”ook on the “same” phenomenon.

**UT-Dallas (University of Texas at Dallas)**: UT-Dallas [55] contains 1540 video clips of 284 people of Caucasian descent walking and conversing. During filming, the subject watched a ten-minute video, which contained scenes from various movies and television programs intended to elicit different emotions in order to capture emotions such as happiness, sadness and disgust.

**RU-FACS (Rochester/UCSD Facial Action Coding System)**: RU-FACS [56] contains 100 subjects that attempted to convince an interviewer he or she is telling the truth. Interviewers were current and former members of the police and FBI.

**MIT (The MIT Media Laboratory, Cambridge MA, USA)**: MIT [57] contains over 25,000 frames scored of 17 drivers that gave their consent to having video and the physiological signals recorded during the drive.

**UA-UIUC (University of Illinois at Urbana-Champaign)**: UA-UIUC [58] contains 28 subjects and one video clip for each subject. First, the subjects could not know that they were being tested for their emotional state. Second, subjects were interviewed after the test to find out their true emotional state for each expression.

**AAI (Adult Attachment Interview)**: The AAI [59] dataset contains 60 subjects from different ethnic groups (European American and Chinese American). The subjects were interviewed and asked to describe the childhood experience. It contains one audiovisual for each subject.

**Smile dataset (Dynamics Of Facial Expression: Normative Characteristics And Individual Differences)**: The Smile dataset [60] contains 195 spontaneous smiles of 95 subjects. Videos were collected throughout a session that included baselines (seated with eyes open) and viewing of film clips.

Overall, the investigated datasets including spontaneous macro-expressions are the majority with 39 instances. The number of subjects included in such datasets ranges fromess than 50 to more than 500. The typical number of subjects is not related with other features, like age range or ethnic diversity or even the amount of data. For instance, the TAVER dataset includes 17 subjects, with an age range between 21 and 38 years and only one ethnicity (Korean); the DISFA dataset comprises 27 subjects with an age ranging between 18 and 50 years and four ethnicities (Asian, Euro American, Hispanic, and African American). Aarge number of subjects does not necessarily correspond to more diversity. For example, the DynEmo dataset with 358 subjects has an age that ranges between 25 and 65 years, and only one ethnicity (Caucasian). That being said, the SEWA dataset with 398 subjects, has an age ranging between 18 and 65 years, and six ethnicities (British, German, Hungarian, Greek, Serbian, and Chinese), and it contains annotations for facialandmarks, acousticow-level descriptors, hand gestures, head gestures, facial action units, verbal and vocal cues, continuously valued valence, arousal andiking/disliking (toward an advertisement), template behaviors, episodes of agreement/disagreement and mimicry episodes. Finally, each dataset handles a different class of emotions, the six basic emotions and neutral (iSAFE) or the six basic emotions and embarrassment and pain (BP4D-Spontaneous), four emotions (ISED) or even one emotion (smile dataset). Some other datasets represent emotions in form of valence and arousal (DEAP, AVEC’14).

### 2.2. Spontaneous and Posed Datasets

We consider herein the spontaneous and the posed datasets due to the fact that we are interested in the spontaneous part of it.

**4DFAB (4D Facial Expression Database for Biometric Applications)**: The 4DFAB [61] dataset includes six posed expressions, spontaneous expressions (anger, disgust, fear, happiness, sadness and surprise), and nine words utterances (puppy, baby, mushroom, password, ice cream, bubble, Cardiff, bob, rope). It contains recordings of 180 subjects captured in four different sessions spanning over a five-year period. This dataset encloses 4D videos of subjects displaying both spontaneous and posed facial behaviors.

**BAUM-1 (Bahcesehir University Multimodal Affective Database-1)**: BAUM-1 [16] contains 31 Turkish subjects and 1,184 multimodal facial video clips. The expressed emotional and mental states consist of happiness, anger, sadness, disgust, fear, surprise, boredom, contempt, confusion, neutral, thinking, concentrating, and bothered.

**MAHNOB Laughter (The MAHNOB Laughter database)**: MAHNOB Laughter [62] contains 22 subjects from 12 different countries and of different origins recorded in 180 sessions. In particular, there are 563 aughter episodes, 849 speech utterances, 51 posedaughs, 67 speech–laughs episodes and 167 other vocalizations annotated in the dataset.

**PICS-Stirling ESRC 3D Face (Psychological Image Collection at Stirling-ESRC project 3D Face Database)**: PICS-Stirling ESRC 3D Face [63] contains 99 subjects, a number of 2D images, video sequences as well as 3D face scans. Seven different expression variations were captured.

**Hi4D-ADSIP (High Resolution 4 Dimensional Database from the Applied Digital Signal and Image Processing Research Centre)**: Hi4D-ADSIP [12] contains 80 subjects from undergraduate students from the Performing Arts Department at the University as well as undergraduate students, postgraduate students and members of staff from other departments. It involves 3360 images/sequences and consists of seven basic facial expressions and further seven facial articulations.

**USTC-NVIE (University of Science and Technology of China (USTC)-Natural Visible and Infrared Facial Expression Database for Expression Recognition and Emotion Inference)**: USTC-NVIE [64] contains 215 subjects, 236 images and six basic expressions. Two kind of facial expressions were recorded: spontaneous expressions induced by the film clips and posed ones obtained by asking the subjects to perform some series of expressions in front of the cameras.

**MMI-V (Induced Disgust, Happiness and Surprise: an Addition to the MMI Facial Expression Database)**: MMI-V [65] contains 25 subjects from different ethnic groups (European, South American, and Asian) recorded in one hour and 32 min of data and 392 segments. Part IV of the dataset was annotated for the six basic emotions and facial muscle actions. Part V of the dataset was annotated for voiced and unvoicedaughter. There are Part IV and Part V because MMI-V dataset was added to the MMI [66] facial expression dataset.

**MMI (The acronym MMI comes from M&M Initiative where the Ms are the initials of the two main authors. Although other colleagues joined the development efforts of the main authors, the acronym remained in use)**: The MMI dataset contains 19 subjects from different ethic groups (European, Asian, or South American), 740 static images sequence of frontal and side view and 848 videos.

**AVLC (The AVLaughterCycle Database)**: AVLC [67] contains 24 subjects from different nationality and ethnic groups (Belgium, France, Italy, UK, Greece, Turkey, Kazakhstan, India, Canada, USA, and South Korea) and 1000 spontaneousaughs elicited by a funny movie and 27 actedaughs.

**IEMOCAP (The Interactive Emotional Dyadic Motion Capture)**: IEMOCAP [68] contains 120 actors (fluent English speakers) recorded in 12 h of audiovisual data, including video, speech, motion capture of faces and text transcriptions. The actors performed selected emotional scripts and also improvised spontaneous spoken communication scenarios to elicit specific types of emotions (happiness, anger, sadness, frustration and neutral state).

**AMI (Augmented Multi-party Interaction)**: The AMI [69] dataset contains a multi-modal set of data consisting of 100 h of meeting recordings, where some of them are naturally occurring, and some others are elicited. In thisatter case, a particular scenario is used where the participants play different roles in a design team, taking a design project from kick-off to completion over the course of a day.

Although we did not discuss posed expressions, we included spontaneous and posed macro-expressions in our survey with 11 datasets. In these categories, the 4DFAB dataset presents an interesting age range that covers infants and elders from 5 to 75 years. Furthermore, the USTC-NVIE dataset presents the highest number of subjects with 215 students. Although MAHNOB Laughter dataset contains an important ethnicity variation (12 different countries and of different origins), its average age is between 27 and 28 years.

### 2.3. In-the-Wild Datasets

In in-the-wild datasets, the human–human interaction results in a spontaneous expression, so that the emotional content and the experimental conditions are uncontrolled.

**RAF-DB (Real-world Affective Faces Database)**: RAF-DB [70] includes thousands of subjects with 30,000 facial images collected from Flickr.

**Aff-Wild2 (Extending the Aff-Wild Database for Affect Recognition)**: The Aff-Wild2 dataset contains videos downloaded from YouTube with 258 subjects from infants and young children to elderly people [71]. It illustrates various ethnicity groups (Caucasian, Hispanic or Latino, Asian, black, and African American), different professions (e.g., actors, athletes, politicians, journalists); as well as changes in head pose, illumination conditions, occlusions and emotions.

**AM-FED+ (An Extended Dataset Affectiva-MIT Facial Expression Dataset)**: In the AM-FED+ [72] dataset, 416 participants from around the world (theirocations are not known) were recruited to watch video advertisements. It contains 1044 videos of naturalistic facial responses to online media content recorded over the Internet.

**AffectNet (Affect from the InterNet)**: AffectNet [73] contains more than 1,000,000 facial images from the Internet of more than 450,000 participants, presenting valence and arousal in eight emotion categories.

**Figure 4:**
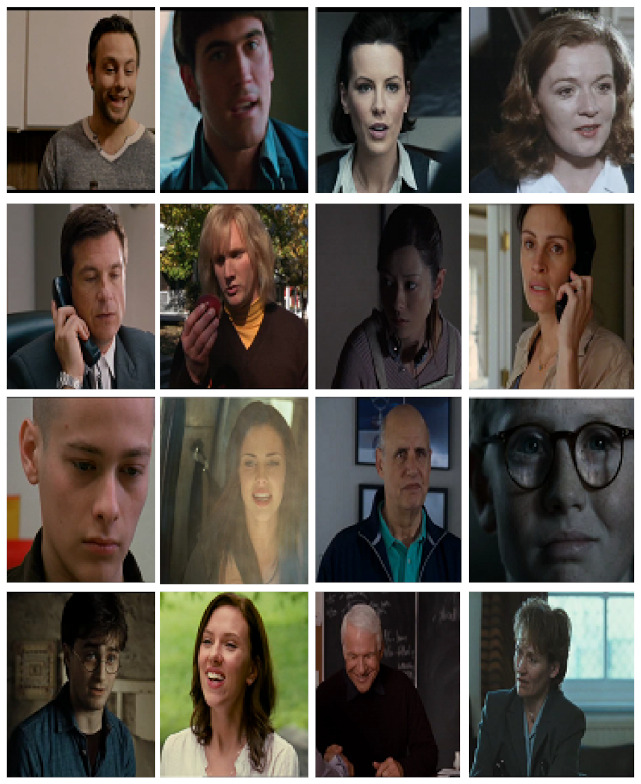
Sample frames from the AFEW-VA dataset in-the-wild.

**AFEW-VA (Database for valence and arousal estimation in-the-wild)**: The AFEW-VA dataset [74] (Figure 4) contains 240 movie actors in a range of age between 8 and 76 years and 600 video clips.

**Aff-Wild (Affectiva-MIT Facial Expression Dataset)**: Within the Aff-Wild dataset [75], more than 500 videos were collected from YouTube, while capturing subjects displaying a number of spontaneous emotions. The data were tagged using emotion-related keywords such as feeling, anger, hysteria, sorrow, fear, pain, surprise, joy, sadness, disgust, love, wrath, contempt, etc.

**EmotioNet (Annotating a million face images in the wild)**: EmotioNet [17] contains one million images of facial expressions downloaded from the Internet, categorized within one of the 23 basic and compound emotion categories. The images have been annotated either with emotion category or with corresponding AUs.

**FER-Wild (Facial Expression Recognition from World Wild Web)**: FER-Wild [15] contains 24,000 Web images from Google, Bing, Yahoo, Baidu and Yandex. These image were categorized in nine categories (no-face, six basic expressions: happy, sad, surprise, fear, disgust, anger, neutral, none, and uncertain). The ’no-face’ category is defined in the following cases: there is no face in the image, there is a watermark on the face, the bounding box was not on the face or did not cover the majority of the face, the face is a drawing, animation, painted, or printed on something else, and the face is distorted beyond a natural or normal shape. The ’no-face’ is defined even if an expression could be inferred. The ’none’ category is defined when the images do not present the six basic emotions or neutral (such as sleepy, bored, tired, etc.). The ’uncertain’ category is defined when the annotators are unsure of the facial expressions.

**Vinereactor (Reactions for vine videos)**: Vinereactor [76] contains 222 mechanical tuckers works filmed with a webcam watching 200 random vine videos from the comedy vine.co channel to get their reactions.

**CHEAVD (Chinese Natural Emotional Audio–Visual Database)**: CHEAVD [77] is extracted from 34 films, two TV series and four other television shows presenting 26 non-prototypical emotional states, including the six basic ones, from 238 speakers.

**HAPPEI (HAPpy PEople Images)**: HAPPEI [78] contains 4886 images downloaded from Flickr of 8500 faces, manually annotated by four humanabelers. The emotions have been categorized according to groupevel happiness intensity (neutral, small smile, large smile, smal laugh, large laugh and thrilled).

**AM-FED (Affectiva-MIT Facial Expression Dataset)**: AM-FED [79] contains 242 facial videos of 242 webcam videos recorded in real world conditions of viewers, from a range of ages and ethnicities, while watching films. It isabeled for frame-by-frameabels for the presence of ten symmetrical FACS action units, four asymmetric (unilateral) FACS action units, two head movements, smile, general expressiveness, feature tracker fails and gender.

**FER-2013 (Facial Expression Recognition 2013 dataset)**: FER-2013 [11] contains 35,685 facial expressions from images queried from the web. Images were categorized based on the emotion shown in the facial expressions (happiness, neutral, sadness, anger, surprise, disgust, fear).

**SFEW (Static Facial Expressions in the Wild)**: SFEW [7] is an extracted dataset (by selecting frames) from the AFEW [10] dataset.

**AFEW (Acted Facial Expressions in the Wild)**: AFEW contains 330 subjects from fifty-four movie DVDs, 1426 sequences, seven emotions (anger, disgust, fear, sadness, happiness, neutral, and surprise) and 1747 numbers of expressions.

**Belfast induced (Belfast Natural Induced Emotion Database)**: The Belfast induced dataset [80] is divided into three tasks: Set 1 tasks contains 114 subjects from undergraduate students and encloses 570 audio-visuals. It is developed as stimuli for research into the individual differences that might influence human abilities to encode and decode emotional signals. Set 2 tasks contains 82 subjects from undergraduate students and postgraduate students or employed professionals, and encloses 650 audio-visuals. It is developed to allow comparison of these new tasks with more traditional film elicitors that had previously been validated for their ability to induce discrete emotions. Set 3 tasks contains 60 subjects from three different ethnic groups (Peru, Northern Ireland) and encloses 180 audio-visuals. It contains variants of the disgust and fear (both active/social) tasks and the amusement (passive/social) task from Set 1. The emotions were self reported by the participants.

**VAM-faces (“Vera Am Mittag”–German TV talk show)**: The VAM-faces [81] dataset consists of 12 h of audio-visual recordings of the German TV talk show “Vera am Mittag”, which were segmented into broadcasts, dialogue acts and utterances. It contains 20 speakers and a set of 1867 images (93.6 images per speaker on average).

**FreeTalk (Tools and Resources for Visualising Conversational-Speech Interaction)**: The FreeTalk [82] dataset contains four subjects from different countries having a conversation in English. It consists of two 90-minute multiparty conversations, and the naturalness of the dialogues is further indicated by the topics of the conversation.

**EmoTV (emotional video corpus: TV interviews (monologue))**: The EmoTV [83] dataset is a corpus of 51 video clips recorded from French TV channels containing interviews. It contains 48 subjects interviewed with a range of 14 emotions classes (anger, despair, doubt, disgust, exaltation, fear, irritation, joy, neutral, pain, sadness, serenity, surprise, and worry).

**BAUM-2 (a multilingual audio-visual affective face database)**: BAUM-2 [13] contains 286 subjects from 122 movies and TV-series result 1047 video clips in twoanguages (Turkish, English). It involves eight emotions (anger, happiness, sadness, disgust, surprise, fear, contempt and neutral). The dataset also provides a set of annotations such as subject age, approximate head pose, emotionabels and intensity scores of emotions.

Overall, the twenty investigated in-the-wild macro-expressions datasets have the highest number of subjects, reaching thousands of subjects in the RAF-DB dataset, the highest diversity of emotions with 23 categories of emotions in EmotioNet, the maximum number of subjects with participants from around the world in the AM-FED+ dataset.

### 2.4. Other Categorizations of Macro-Expression Datasets

In the following, we propose other categorizations for the spontaneous and in-the-wild datasets. One way is that of considering the different ways the data have been collected:

- In *spontaneous* datasets, unlike posed datasets, where participants are asked to perform an emotion, subjects’ emotions are stimulated. For example, in [9], face expressions were captured when volunteers were asked to watch a few stimulant videos. In a similar way, in [43], participants were shown fragments of movies and pictures. In [31], emotional videos were used for each emotion, and in the dataset investigated in [14], combined interviews, planned activities, film watching, cold pressor, test/social challenge and Olfactory stimulation were explored. In [42], participants were told to change character when they got bored, annoyed or felt they had nothing more to say to the character. The dataset proposed in [49] collected conversational speech, and the work in [51] had been based on a conversation between two people in which one paysittle or no attention to the meaning of what the other says and chooses responses on the basis of superficial cues. In [50], participants were from a clinical trial for treatment of depression, however, in [27], the participant has a dialogue script with vignettes for each emotional category. In [38], subjects had performed a human–computer interaction task, similarly to the work of [39], where natural conversations between pairs of people were investigated. In [59], subjects were interviewed and asked to describe the childhood experience, and in [56], subjects tried to convince the interviewers they were telling the truth. In [48], subjects had described neutral photographs, played a game of Tetris, described the game of Tetris and solved cognitive tasks. Differently, in [57], a driver was recorded during the drive, and the work of [52] presented an interaction from TV chat shows and religious programs and discussions between old acquaintances. In [53], participants were playing a game in which one person has to explain to the other using gestures and body movement a ‘taboo’ concept or word.
- Within the framework of *in-the-wild* datasets, the collected data come from movies [10,13], films, TV plays, interviews and talk shows [77,81,83], videos downloaded from Youtube [71], images and videos from the Internet [17,73,84] as well as from Flickr [70,78].

Most of the datasets have classified emotions into the six basic categories (angry, disgust, fear, happy, sad, surprise) [7,64,65,66], with some datasets adding the neutral one [9,10,11]. There are also datasets that further extended the basic six plus neutral expression model with one additional expression, like pain [12], or contempt [13]. Other datasets added more expressions, like happiness or amusement/sadness/surprise or startle/embarrassment/fear or nervous/pain/anger or upset/disgust [14]. Actually, a variety of expressions can be found in the existing datasets over those indicated above. For example, there are twenty-three categories of emotion in [17] according to [85]; nine categories (no-face, six basic expressions, neutral, none, and uncertain) in [15]; thirteen emotional and mental states are included in [16], where the six basic emotions plus boredom and contempt are complemented with some mental states (i.e., confusion, neutral, thinking, concentrating, and bothered); four emotions (sadness, surprise, happiness, and disgust) are given in [31]; with only one emotion (smile) being included in [60,79]. The Valence and Arousal expression model was instead followed in [35,41,73,75]. We note some datasets that also included Action Unit (AU) annotations. For instance, the EmotioNet [17] and DISFA [36] datasets have 12 AUs annotations, and in the CASME [86] dataset, AUs are coded by two coders based on Ekman’s study. Table 2 groups the datasets according to the different ways emotions are categorized.

It is worth mentioning that some datasets contain 3D scans of expressive faces. For example, 4DFAB [61] contains 3D faces (over 1,800,000 3D meshes), and PICS-Stirling ESRC 3D Face Database [63] presents 3D face scans along with 2D images and video sequences. Likewise, CAM3D [45] is a 3D multimodal corpus dataset, and B3D(Ac) [46] dataset presents facial expressions in dynamic 3-D face geometries. Likewise, BP4D+ [25] contains high-resolution 3D dynamic imaging with a variety of sensors of the face, 4D CCDb [32] is a 4D (3D Video) audio-visual dataset, BP4D-Spontaneous [14] is a 3D video dataset of spontaneous facial expressions, and Hi4D-ADSIP [12] presents a comprehensive 3D dynamic facial articulation dataset.

In what follows, we propose some other categorizations for macro-expression datasets:

- **Number of subjects**: Table 3 presents a classification of macro-expression datasets according to the number of subjects. Most of the datasets containess than 50 subjects, with just few datasets containing more than 500 subjects. The number of subjects can reach more than thousands, if the expressions are spontaneous or in-the-wild.
- **Age variation**: There are many age ranges in macro-expression datasets. Most of the datasets include subjects in a relatively small range (from 18 to30 years), namely TAVER, RAVDESS, GFT, MAHNOB Mimicry, BP4D-Spontaneous, MAHNOB Laughter, DEAP, USTC-NVIE, MMI-V, AvID, AVIC, ENTERFACE, UT-Dallas, RU-FACS, UA-UIUC, AAI, iSAFE, and ISED. Some other datasets have a moderate range (18–60), including EB+, SEWA, BP4D+ (MMSE), BAUM-1, BioVid Emo, 4D CCDb, AVEC’14, DISFA, AVEC’13 AViD-Corpus, CCDb, DynEmo, SEMAINE, MAHNOB-HCI, Hi4D-ADSIP, CAM3D, B3D(AC), CK+, VAM-faces, and MM. Few datasets contain children, including CHEAVD, 4DFAB, BAUM-2, AFEW-VA, AFEW, and Aff-Wild2. However, child facial expressions were mixed within adult expression samples without differentiating them based on age or age group. On the other hand, in the CHEAVD dataset, the participants were divided into six groups of ages, and in the 4DFAB dataset, the age distribution includes five categories, with infants being in the 5–18 category. However, the datasets did not take into consideration the difference of the facial expressions according to the age.
- **Frame per second (FPS)**: In macro-expression analysis, the number of FPS is relevant depending on the application context. In the following datasets, the number of FPS is smaller or equal to 20: TAVER, AM-FED+, and AM-FED. Instead, the number of FPS is greater than 50 for the 4DFAB, 4D CCDb, MAHNOB-HCI, Hi4D-ADSIP, FreeTalk, iSAFE, and ISED datasets. Theargest number of FPS, equal to 120, is reached in the IEMOCAP dataset, which makes it a relevant source for studying macro expressions.
- **Ethnicity**: The existing macro-expression datasets contain various ethnicities such as Latino (EB+, 4DFAB, Aff-Wild2, BP4D+, RU-FACS), Hispanic (EB+, 4DFAB, Aff-Wild2, BP4D+, BP4D-Spontaneous, DISFA), White (EB+, BP4D+), African (EB+, Aff-Wild2, BP4D+, BP4D-Spontaneous, DISFA), Asian (EB+, 4DFAB, Aff-Wild2, BP4D+, BP4D-Spontaneous, DISFA, CAM3D, MMI-V, AVIC, MMI, RU-FACS, iSAFE), and Caucasian (4DFAB, Aff-Wild2, RAVDESS, DynEmo, CAM3D, UT-Dallas). However, some datasets contain participants from around the world or randomly selected (RAF-DB, AM-FED+, GFT, AffectNet, AFEW-VA, EmotioNet, AM-FED, AFEW, FreeTalk).
- **Amount of data**: Here, the main distinction is between datasets that include images;ike EB+, TAVER, Aff-Wild2, AM-FED+, AFEW-VA, SEWA, Aff-Wild, BAUM-1, BioVid Emo, Vinereactor, CHEAVD, 4D CCDb, OPEN-EmoRec-II, AVEC’14, RECOLA, AM-FED, AVEC’13, CCDb, DynEmo, DEAP, AFEW, Belfast induced, MAHNOB-HCI, UNBC-McMaster, CAM3D, B3D(AC), UT-Dallas, EmoTV, UA-UIUC, and AAI; and datasets that instead comprise videos;ike RAF-DB, AffectNet, EmotioNet, FER-Wild, HAPPEI, FER-2013, SFEW, USTC-NVIE, iSAFE, and ISED.

### 2.5. Current Challenges and Future Perspectives

Up to this point, we have described and discussed characteristics of macro-expressions related datasets. Research on macro-expression recognition has evolved significantly in theast few years, while reaching saturated performance onab-controlled, small-sized datasets, and the significant advancement of recognition methods call for new challenges.

The number of datasets in-the-wild is stillimited compared to spontaneous datasets. Indeed, most of the spontaneous datasets contain few subjects unlike in-the-wild ones which contain many more subjects that can reach thousands as in the RAF-DB dataset [70]. The variation in ethnicity in spontaneous datasets ranges between one and six different ethnic groups in each dataset, while the captured subjects in in-the-wild datasets are from around the world. The ethnic element is important because thearger the diversity, the more interesting the dataset can be, and this is due to the fact that there are differences in facial expression depending on ethnicity [87]. For instance, in [88], authors have found “aower mean recognition accuracy of Caucasian faces among African and Japanese subjects than among Europeans and Americans subjects”. The age ranges between infants and elderly, however, few datasets contain children; e.g., CHEAVD, BAUM-2, AFEW-VA, AFEW, Aff-Wild2, and few datasets contain elders, e.g., EB+, 4DFAB, Aff-Wild2, BAUM-2, and BP4D+(MMSE); the rest of the datasets have an average of 20-30 years. The age variance is important due to the fact that child as elders’ facial expressions can actually be different compared to adult expressions. It could be interesting to have datasets with aarger number of subjects in order to have a wider diversity in the ethnicity and age range. This would also help in including more diversity in the way expressions are performed. More in general, increasing the number of emotion categories, going beyond the six basic emotions, is a further direction for the facial expression datasets in the next years.

## 3. Micro-Expression Datasets

Micro-expressions are defined as facial expressions thatast for only a very short time period. They are shown as the result of an emotional response that activates, in an innate way, both voluntary and involuntary expressions of the face that conflict one with the other. As a result, the individual shows the true expression just for a very short time interval, which is then followed by a false expressive reaction [89]. Overall, studies have shown that this occurs when a part of our brain (i.e., the amygdala) responds to the emotional stimuli experienced by an individual in an appropriate way by showing a facial expression, but then the individual consciously decides to cancel that expression/emotion. In fact, while macro-expressionsasts from 0.5 to 4 s [89], a micro-expression normally has a duration ofess than half of a second [90]. Due to this very short duration, and differently from macro-expressions, micro-expressions cannot be controlled and so they are very difficult or even impossible to hide. The fact that micro-expressions are expressive reactions thatast just some fractions of seconds implies that they are best captured by high-speed cameras [91]. According to the works of Ekman, micro-expressions can be categorized into the seven universal emotions: disgust, anger, fear, sadness, happiness, contempt, and surprise. Ekman himself subsequently expanded thisist including a range of positive and negative emotions; namely, amusement, embarrassment, anxiety, contentment, guilt, pleasure, pride, relief, and shame (not all of them are encoded by facial muscles). Herein, we summarize the existing datasets for micro-expression analysis while following a similar organization as the one that we proposed for macro-expression ones. We start by describing the spontaneous datasets, then we discuss the in-the-wild ones. We also put in other evidence relevant features for these datasets. Indeed, comparing the abundance of macro-expression datasets with theimited number of micro-expression ones, it evidently comes the unbalanced proportion, suggesting that the research on micro-expression recognition isess developed than that for macro-expressions.

### 3.1. Spontaneous Datasets

We have identified nine datasets in theiterature that have been used with a certain frequency for the analysis of micro-expressions. They have been acquired with quite heterogeneous devices and proposing different evaluation protocols.

**SAMM (Spontaneous Micro-facial Movement)**: The SAMM [92] dataset contains 32 participants from 13 different ethnic groups and 159 samples with seven emotions (contempt, disgust, fear, anger, sadness, happiness and surprise).

**CAS(ME)2 (Chinese Academy of Sciences Micro-expression–A Database for Spontaneous Macro-expression and Micro-expression Spotting and Recognition)**: This dataset was proposed in [93]. It contains 22 subjects and 53 samples with four emotions (positive, negative, surprise, and others).

**Silesian deception dataset**: The Silesian deception dataset [94] includes 101 students of the third year and the fourth year at the Faculty of Automatic Control, Electronics and Computer Science. This comprises 101 samples and frame annotations, including eye closures (eye movements), gaze aversion and micro-tensions.

**CASME II (Improved Spontaneous Micro-Expression)**: CASME II [95] contains 247 samples selected from 3000 elicited facial movements, from 26 participants presenting five classes of emotions (happiness, disgust, surprise, repression and others).

**CASME (The Chinese Academy of Sciences Micro-expression)**: This dataset was introduced in the work of [86]. It includes seven emotions (tense and repression in addition to the basic emotions), from 35 participants, with only 19 of them considered as valid. It contains 195 micro-expressions (selected from more than 1500 elicited facial movements) divided into two classes (*A* and *B*). The class *A* includes 100 samples, while 95 samples are comprised by the class *B*. The facial expressions were recorded in two different environmental configurations by using two different cameras: the first one with naturalight and 1280×720 resolution, and the second one with two LEDights and a resolution of 640×480.

**SMIC-E (Extended version of SMIC–Spontaneous Micro-expression)**: SMIC-E [96] contains 32 participants filmed under different conditions and timings. There are 16 participants who were recorded with a High-Speed (HS) camera (PixeLINK PL-B774U, 640×480) at 100 fps. Theongest micro-expression clips have a duration of 50 frames for a total of 167 samples. Other eight participants were recorded with a normal visual camera (VIS) at 25 fps, in addition to the high-speed camera. In this case, theongest micro-expression clips have 13 frames for a total of 71 samples. The same number of samples were included in the third part of the dataset captured with a near-infrared (NIR) camera at 25 fps in addition to the high-speed camera. In this case, theongest micro-expression clips have 13 frames, such that all of them involve three emotions (positive, negative and surprise).

**SMIC (Spontaneous Micro-expression)**: In the SMIC [97] dataset, six subjects were recorded, with a camera at 100 fps, while watching 16 films. The acquisitions were performed while instructing the participants to suppress their facial expressions whilst carefully watching the clips. The proposed experiments aim to guess which film clip the subject is watching byooking at his/her face. The acquisitions involve five emotions (disgust, fear, happiness, sadness and surprise) and 77 spontaneous micro-expressions.

**Canal9 (A Database of Political Debates for Analysis of Social Interactions)**: This dataset was recorded by the Canal 9ocal TV station and broadcast in Valais, Switzerland [98]. It includes 190 participants, collected during 70 debates for a total of 43 h and ten minutes of material, involving 24 sequences of micro-expressions.

**YorkDDT (York Deception Detection Test–University of York)**: In the YorkDDT dataset [99], the micro-expression sequences of nine subjects were segmented andabeled as truthful/deceptive and/or emotional/non-emotional. This resulted into 18 samples from 20 videos for a Deception Detection Test (DDT) and two emotion classes.

### 3.2. In-the-Wild Datasets

We were able to identify just one micro-expression accessible datasets acquired in in-the-wild conditions.

**MEVIEW (MicroExpression VIdEos in-the-Wild)**: The MEVIEW [84] dataset contains 31 videos of 16 subjects from poker games and TV interviews downloaded from the Internet. It includes macro- and micro-expressions while illustrating five emotions.

### 3.3. Other Categorizations of Micro-Expression Datasets

We have observed that annotations in micro-expression datasets are classified either with onset (start), apex (peak), offset (end) of the emotion, self-reported, or facial muscle contraction. For example, the frame annotations in SAMM [92], CAS(ME)2 [93], CASME II [95], and CASME [86] are onset, offset, apex; while in the MEVIEW dataset [84], they are onset and offset. Differently, the Silesian deception dataset [94] is annotated with eye closures, gaze aversion and micro-tensions. The annotation of micro-expression video clips in the SMIC-E [96] and the SMIC [97] datasets have been obtained according to participants’ self-reported emotions. However, in the Canal9 dataset [98], the annotations are manual speaker segmentation, role, agreement and disagreement, automatic speaker segmentation, manual shot segmentation, automatic shot segmentation, manual shot classification and manual identification of participants in personal shots. In a similar way to what we presented for macro-expressions, we herein organize the micro-expression datasets according to different categorizations: number of subjects, FPS, amount of data/frames, samples, FACs coded, lights and resolution.

- **Number of subjects**: Table 4 presents a classification of micro-expression datasets according to the number of enrolled subjects. We classify the datasets according to the fact they involveess than 50 participants or more than 100 participants.
- **Frame per second (FPS) and resolution**: Due to the importance of the FPS rate in the detection of micro-expression datasets, we have found that the number of FPS reaches the value of 200 in both the SAMM and the CASME II datasets, which is a higher number than that used in macro-expression datasets. In the following datasets, the number of FPS is equal or greater than 100: Silesian deception, CASME, SMIC-E HS, and SMIC. There are also micro-expression datasets, where the number of FPS is smaller than 50 as for CAS(ME)2, MEVIEW, SMIC-E VIS, SMIC-E NIR, and YorkDDT. To help capture more subtle facial movements, a higher number of FPS and resolution is needed. As best as we know, the highest resolution available for micro-expressions datasets is 2040×1088 pixels as presented by the SMM dataset; and theowest resolution set, instead, is equal to 320×240 as contained in the YorkDDT dataset. For the rest of the micro-expression datasets, the resolution is set to 640×480 in the CAS(ME)2, Silesian deception, CASME II, CASME, SMIC-E, and SMIC datasets.
- **Amount of data and samples**: Unlike macro-expression datasets, most of the micro-expression datasets contain videos. The major difference between micro- and macro-expressions resides in the number of samples and/or the number of micro-expressions. We classify the datasets according to whether they containess than 50 samples as in MEVIEW, Canal9 and YorkDDT, between 50 and 100 samples as in CAS(ME)2, SMIC-E VIS, SMIC-E NIR and SMIC, or between 100 and 200 samples as in SAMM, Silesian deception, CASME and SMIC-E HS. The CASME II dataset includes 247 samples.
- **Lights**: Micro-expression datasets propose severalightning conditions. Fourights have been used in both the CASME II and the SMIC-E datasets, while twoights were performed for SAMM and CAS(ME)2 and in the second class of CASME.

### 3.4. Current Challenges and Future Perspectives

Apart from having only one dataset with in-the-wild expressions, i.e., the MEVIEW dataset [84], there is still work to do for micro-expression datasets. First, the number of subjects is still small, not exceeding 200 subjects. Second, the age range isimited, and most of the subjects are in their twenties. In fact, we did not find children or elders included in the micro-expressions in order to have more diversity. The emotion variance is alsoimited, with only two datasets including seven emotions; i.e., SAMM [92] and CASME [86], while the rest present a number of emotions between two and five. It is worth noting that almost all the micro-expression datasets were created in an indoor environment. It could be interesting to have more in-the-wild micro-expression datasets with aarger number of subjects and a wider age range, so as to include children and elders, and have more emotion variation. The spontaneous macro-expression datasets areisted in Table 5 and Table 6.

## 4. Applications

Datasets vary in the number of participants, head pose, age, video resolution, number of frames, number of subjects, and context. In this section, we comment about the most used datasets in the state-of-the-art and the main contexts of applications according to the most recent works.

### 4.1. Medical Applications

Detecting signs of depression, pain or even diagnosing rare conditions of disease can be identified based on specific features that are derived just looking to the face, like heart beat, skin texture, and skin temperature. Some datasets include those features to detect emotions, but they can be used to detect health signs. Furthermore, GET [28] is a dataset of group formation (Healthy social drinkers), and the BioVid Emo [30] dataset combines psycho-physiological signals with video signals for discrete basic emotions. The OPEN_EmoRec_II [34] dataset includes physiology annotations (SCL, respiration, BVP, EMG Corrugator supercilii, EMG Zygomaticus Major). Likewise, the MAHNOB-HCI [43] dataset provides physiological sensors measuring ECG, EEG (32 channels), respiration amplitude, and skin temperature. Nevertheless, the UNBC-McMaster or the UNBC Shoulder Pain Archive (SP) [44] dataset has spontaneous facial expressions relating to genuine pain, where participants were self-identified as having a problem with shoulder pain. The DD [50] dataset was created with participants from a clinical trial for treatment of depression. It is worth noting that, in addition to the above mentioned pain and depression datasets, there are studies imaging facial expressions of patients with Alzheimer’s disease [100], Parkinson’s [101], schizophrenia [102], and autism [103]. However, most of the datasets from these studies are protected by privacyaws such as HIPPAaws and hence are not shared publicly.

### 4.2. Smart Driving Applications

Driving a car has becomeike interacting with a social robot. Therefore, the emotional status of drivers is mandatory to build smart driving applications. In the MIT [57] dataset drivers were recorded, while their physiological signals are recorded during the drive (consent to the driving monitoring is collected).

### 4.3. Social Marketing Applications

To predict buyers practices, commercial applications tend to watch the reactions of people to ads, such as in the AM-FED+ [72] dataset and in the AM-FED [79] dataset, where subjects were watching amusing super bowl commercials.

### 4.4. Human Computer Interactions

Some datasets present the facial expressions when participants are performing a human–computer interaction. For example, the iSAFE [9] dataset is an Indian dataset where volunteers were watching a few stimulant videos, and in the AVEC’13 dataset as well as the AViD-Corpus [38] dataset, subjects were recorded using a webcam and a microphone.

## 5. Discussion and Conclusions

In this work, we have proposed a survey of macro- and micro-expressions facial datasets. Since it is difficult to classify all reported datasets due to their difference from each other in terms of participant’s age and ethnicity, number of subjects and amount of data, we have divided them according to their content as *spontaneous* or *in-the-wild*. Spontaneous and in-the-wild expressions are much more difficult to classify in terms of recognition rate than posed expressions. Since macro-expression datasets take theargest part of this survey with 70 datasets, we have divided the datasets as spontaneous, in-the-wild, and we have also included datasets that present both spontaneous and posed expressions. Then, we have classified them according to the number of subjects, the age variation, the rate of frames per second (FPS), the ethnicity, and the amount of data. Regarding micro-expressions, in general, the research on this topic isess developed when compared to the results available for macro-expressions. As a consequence, a muchower number of micro-expression datasets do exist, with only one micro-expression dataset captured in-the-wild. The survey also reports some applications where the discussed datasets have been investigated. In particular, we have identified and exemplified the use of expression datasets in four different contexts. One interesting aspect that emerges from this analysis is the idea of considering the timeapse in the acquisition of subjects, so that an emotion variation can be observed across elapsed time. Actually, only two datasets took the timeapse into consideration: the AViD-Corpus dataset [38], where two different recordings captured with a two-weeks interval are included, and the Smile dataset [60], where the interval between two smiles acquisitions is of one year. Moreover, several factors related to the dataset quality and characteristics may influence the facial expression recognition and make it a challenging problem, such as the data size, age and gender of subjects, recording environment and devices. In addition to these factors, personality or mood of the subjects are external factors that may alter the FER process. Indeed, some datasets give advance information about the experimental procedure for the subjects (USTC-NVIE), while others gave no instructions to the subjects on how they should react and what was the purpose of the study (MAHNOB). In some cases, there is no detailed description on how the dataset videos were selected by collectors or psychologists. Besides, several other factors, such as the recording environment, the recording distance, shooting angle, and more importantly the order setup for recording different emotions (e.g., to reduce the influence of the previous emotion, neutral videos were shown to subjects in USTC-NVIE), affect the quality of collected data and consequently represent a challenge for FER. Moreover, there is an imbalanced attribution of emotions in most of the datasets: for example, in the ISED dataset [31] the number of clips is 227 for happiness, 73 for surprise, 48 for sadness, and 80 for disgust; in the BAUM-2 dataset [13], there are 248 happiness clips, 173 anger, 137 sadness, 51 disgust, 152 surprise, 68 fear, 49 contempt, 169 neutral; in the AFEW dataset [10], 194 anger, 123 disgust, 156 fear, 165 sadness, 387 happiness, 257 neutral, and 144 surprise clips. Combining together more than one dataset can be a plausible way to solve thisack of balance.

In summary, we can draw some final considerations about the data currently available for facial expression research. For both macro- and micro- expressions, we think a desirable trend is that of introducing new in-the-wild datasets. This has the clear advantage of providing real-world data, while also scaling toarge amount of different subjects and instances. The differences in the ambient where subjects are immersed and the real-life contexts can add the needed variability in the data that can improve the neural network capability of generalizing to unseen scenarios. This is more evident for macro-expression datasets, while only one micro-expression dataset is going in this direction. We can hypothesize that more micro-expression datasets acquired in-the-wild will appear in the next few years. For macro-expression datasets, another trend that we think could be fruitful is that of providing an ampler spectrum of expression annotations. Though the Ekman’ six expression model remains useful for a coarse expression analysis, having additional expressions, while also including mental and emotional states could provide a more comprehensive view of the expression reactions of captured individuals. In this respect, continuous models, like the valence–arousal one, appear promising and as the possible future standard annotations for macro-expression datasets. In the case of micro-expressions, the six-expressions model remains the reference one, while alternative annotation proposals have not emerged yet in a consolidated way. As a result, most of the micro-expression datasets have proposed specific annotations. 

## Figures and Tables

**Table 1:** Proposed categorization of macro- and micro-expression datasets.

Macro- and Micro-Expressions Facial Datasets			
Macro-Expression Datasets		Micro-Expression Datasets	
Spontaneous	In-the-wild	Spontaneous	In-the-wild
EB+, TAVER, RAVDESS, GFT, SEWA, BP4D+ (MMSE), BioVid Emo, 4D CCDb, MAHNOB Mimicry, OPEN-EmoRec-II, AVEC’14, BP4D-Spontaneous, DISFA, RECOLA, AVEC’13, CCDb, DynEmo, DEAP, SEMAINE, MAHNOB-HCI, UNBC-McMaster, CAM3D, B3D(AC), CK+, AvID, AVIC, DD, SAL, HUMAINE, EmoTABOO, ENTERFACE, UT-Dallas, RU-FACS, MIT, UA-UIUC, AAI, Smile dataset, iSAFE, ISED	RAF-DB, Aff-Wild2, AM-FED+, AffectNet, AFEW-VA, Aff-Wild, EmotioNet, FER-Wild, Vinereactor, CHEAVD, HAPPEI, AM-FED, FER-2013, AFEW, Belfast induced, SFEW, VAM-faces, FreeTalk, EmoTV, BAUM-2	SAMM, CAS(ME)2, Silesian deception, CASME II, CASME, SMIC-E, SMIC, Canal9, YorkDDT	MEVIEW

**Table 2:** Classification of macro-expression datasets according to their content.

Expression Representation	Macro-Expression Datasets
Six basic expressions	MMI, USTC-NVIE, MMI-V, SFEW
Six basic expressions + neutral	iSAFE, AFEW, FER-2013
Six basic expressions + neutral, pain	Hi4D-ADSIP
Six basic expressions + neutral, contempt	BAUM-2
Six basic expressions (happiness or amusement, sadness, surprise or startle, fear or nervous, anger or upset, disgust) + embarrassment, pain	BP4D-Spontaneous
23 categories of emotion	EmotioNet
Nine categories of emotions (no-face, six basic expressions, neutral, none, and uncertain)	FER-Wild
13 emotional and mental states (six basic emotions plus boredom and contempt plus mental states, confusion, neutral, thinking, concentrating, and bothered)	BAUM-1
Four emotions (sadness, surprise, happiness, and disgust)	ISED
One emotion (smile)	AM-FED, Smile dataset
Valence–arousal	AffectNet, DEAP, Aff-Wild, AVEC’14

**Table 3:** Classification of macro-expression datasets according to their number of subjects.

Number of Subjects	Macro-Expression Datasets
≤50	TAVER, RAVDESS, BAUM-1, OPEN-EmoRec-II, BP4D-Spontaneous, DISFA, RECOLA, CCDb, MAHNOB Laughter, DEAP, SEMAINE, MAHNOB-HCI, UNBC-McMaster, CAM3D, B3D(AC), MMI-V, AVLC, AvID, AVIC, VAM-faces, ENTERFACE, MMI, MIT, EmoTV, UA-UIUC, 4D CCDb, FreeTalk, IEMOCAP, SAL, iSAFE, ISED
∈[50, 100]	GFT, SEWA, BioVid Emo, MAHNOB Mimicry, AVEC’14, PICS-Stirling ESRC 3D Face Database, Belfast induced (Set2 and Set3), Hi4D-ADSIP, DD, RU-FACS, AAI, Smile dataset
∈[100, 250]	EB+, 4DFAB, AFEW-VA, BP4D+ (MMSE), Vinereactor, CHEAVD, AM-FED, Belfast induced (Set1), USTC-NVIE, CK+
∈[250, 500]	SFEW, Aff-Wild2, AM-FED+, BAUM-2, AVEC’13 AViD-Corpus, DynEmo, AFEW, UT-Dallas
≥500	RAF-DB, AffectNet, Aff-Wild, EmotioNet, FER-Wild, FER-2013, HAPPEI, HUMAINE

**Table 4:** Classification of micro-expression datasets according to their number of subjects.

Number of Subjects	Micro-Expression Datasets
≤50	SAMM, CAS(ME)2, MEVIEW, CASME II, CASME, SMIC-E, SMIC, YorkDDT
≥100	RAF-DB, AffectNet, Aff-Wild, EmotioNet, FER-Wild, FER-2013, HAPPEI, HUMAINE

**Table 5:** Macro-expressions datasets. The columns report: the dataset name (Dataset); the number of subjects; the range of subjects’ age (Age); the number of frames captured per second (FPS); ethnicity; and the amount of data/frames. In the table cells, a ‘-’ indicates that no information is available, while a ‘*’ following the dataset name indicates that the data is publicly available.

Dataset	Year	Number of Subjects	Age	FPS	Ethnicity	Amount of Data/Frames
**EB+** [24]	2020	200	18–66	25	Five ethnicities (Latino/Hispanic, White, African American, Asian, and Others)	1216 videos, with 395 K frames in total
**iSAFE** [9]	2020	44	17–22	60	Two ethnicities (Indo-Aryan and Dravidian (Asian))	395 clips
*RAF-DB* * [70]	2019	thousands	-	-	The images URLs were collected from Flickr	30,000 facial images
**TAVER** * [26]	2019	17	21–38	10	One ethnicity (Korean)	17 videos of 1–4 mn
4DFAB* [61]	2018	180	5–75	60	Three ethnicities (Caucasian (Europeans and Arabs), Asian (East-Asian and South-Asian) and Hispanic/Latino)	Two million frames. The vertex number of reconstructed 3D meshes ranges from 60 k to 75 k
*Aff-Wild2* * [71]	2018	258	infants, young and elderly	30	Five ethnicities (Caucasian, Hispanic or Latino, Asian, black, or African American)	Extending it with 260 more subjects and 1,413,000 new video frames
**RAVDESS** * [27]	2018	24	21–33	30	(Caucasian, East-Asian, and Mixed (East-Asian Caucasian, and Black-Canadian First nations Caucasian))	7356 recordings composed of 4320 speech recordings and 3036 song recordings
*AM-FED+* * [72]	2018	416	-	14	Participants from around the world	1044 videos of naturalistic facial responses to online media content recorded over the Internet
**GFT** * [28]	2017	96	21–28	-	Participants were randomly selected	172,800 frames
AffectNet* [73]	2017	450,000	average age 33.01 years	-	More than 1,000,000 facial images from the Internet	1,000,000 images with facialandmarks. 450,000 images annotated manually
AFEW-VA* [74]	2017	240	8–76	-	Movie actors	600 video clips
SEWA* [29]	2017	398	18–65	20–30	Six ethnicities (British, German, Hungarian, Greek, Serbian, and Chinese)	1990 audio-visual recording clips
**BP4D+ (MMSE)** [25]	2016	140	18–66	25	Five ethnicities (Latino/Hispanic, White, African American, Asian, and Others)	1.4 million frames. Over 10TB high quality data generated for the research community
*Aff-Wild* * [75]	2016	500	-	-	-	500 videos from YouTube
*EmotioNet* * [17]	2016	1,000,000	-	-	One million images of facial expressions downloaded from the Internet	Images queried from web: 100,000 images annotated manually, 900,000 images annotated automatically
*FER-Wild* * [15]	2016	24,000	-	-	-	24,000 images from web
***BAUM-1*** * [16]	2016	31	19-65	30	One ethnicity (Turkish)	1184 multimodal facial video clips contain spontaneous facial expressions and speech of 13 emotional and mental states
**BioVid Emo** * [30]	2016	86	18–65	-	-	15 standardized film clips
*Vinereactor* * [76]	2016	222	-	web-cam	Mechanical tuckers	6029 video responses from 343 unique mechanical truck workers in response to 200 video stimulus. Total number of 1,380,343 video frames
CHEAVD * [77]	2016	238	11–62	25	-	Extracted from 34 films, two TV series and four other television shows. In the wild
**ISED** * [31]	2016	50	18–22	50	One ethnicity (India)	428 videos
**4D CCDb** * [32]	2015	4	20–50	60	-	34 audio-visuals
**MAHNOB Mimicry** * [33]	2015	60	18–34	25	Staff and students at Imperial College London	Over 54 sessions of dyadic interactions between 12 confederates and their 48 counterparts
**OPEN-EmoRec-II** * [34]	2015	30	Mean age: women 37.5 years; men 51.1 years	-	-	Video, audio, physiology (SCL, respiration, BVP, EMG Corrugator supercilii, EMG Zygomaticus Major) and facial reactions annotations
*HAPPEI* * [78]	2015	8500 faces	-	-	-	4886 images.
**AVEC’14** * [35]	2014	84	18–63	-	German	300 audio-visuals
*BAUM-2* * [13]	2014	286	5–73	-	two ethnicities (Turkish, English)	1047 video clips
**BP4D-Spontaneous** * [14]	2013	41	18–29	25	four ethnicities (Asian, African-American, Hispanic, and Euro-American)	368,036 frames
**DISFA** * [36]	2013	27	18–50	20	four ethnicities (Asian, Euro American, Hispanic, and African-American)	130,000 frames
**RECOLA** * [37]	2013	46	Mean age: 22 years, standard deviation: three years	-	four ethnicities (French, Italian, German and Portuguese)	27 videos
*AM-FED* * [79]	2013	242	Range of ages and ethnicities	14	Viewers from a range of ages and ethnicities	168,359 frames/242 facial videos
*FER-2013* * [11]	2013	35,685	-	-	-	Images queried from web
**AVEC’13 (AViD-Corpus)** * [38]	2013	292	18–63	30 one ethnicity (German)	340 audio-visuals	
**CCDb** * [39]	2013	16	25–56	-	All participants were fully fluent in the Englishanguage	30 audio-visuals
***MAHNOB Laughter*** * [62]	2013	22	Average age: 27 and 28 years	25	12 different countries and of different origins.	180 sessions 563aughter episodes, 849 speech utterances, 51 posedaughs, 67 speech–laughs episodes and 167 other vocalizations annotated in the dataset
**DynEmo** * [40]	2013	358	25–65	25	One ethnicity (Caucasian)	Two sets of 233 and 125 recordings of EFE of ordinary people
***PICS-Stirling ESRC 3D Face Database*** * [63]	2013	99	-	-	-	2D images, video sequences and 3D face scans
**DEAP** * [41]	2012	32	19–37	-	Mostly European students	40 one-minuteong videos shown to subjects
*AFEW* * [10]	2012	330	1–70	-	Extracted from movies	1426 sequences withength from 300 to 5400 ms. 1747 expressions
**SEMAINE** * [42]	2012	24	22–60	-	Undergraduate and postgraduate students	130,695 frames
**Belfast induced** * [80]	2012	Set1: 114	Undergraduate students	-	undergraduate students	570 audio-visuals
		Set2: 82	Mean age of participants 23.78	-	Undergraduate students, postgraduate students or employed professionals	650 audio-visuals
		Set3: 60	age of participants 32.54	-	(Peru, Northern Ireland)	180 audio-visuals
**MAHNOB-HCI** * [43]	2012	27	19–40	60	Different educational background, from undergraduate students to postdoctoral fellows, with different English proficiency from intermediate to native speakers	756 data sequences
***Hi4D-ADSIP*** * [12]	2011	80	18–60	60	Undergraduate students from the Performing Arts Department at the University. Undergraduate students, postgraduate students and members of staff from other departments	3360 images/sequences
**UNBC-McMaster (UNBC Shoulder Pain Archive (SP))** * [44]	2011	25	-	-	Participants were self-identified while having a problem with shoulder pain	48,398 frames/200 video sequences
**CAM3D** * [45]	2011	16	24–50	25	Three ethnicities (Caucasian, Asian and Middle Eastern)	108 videos of 12 mental states
*SFEW* * [7]	2011	95	-	-	-	700 images: 346 images in Set 1 and 354 images in Set 2
**B3D(AC)** * [46]	2010	14	21–53	25	Native English speakers	1109 sequences, 4.67 song
***USTC-NVIE*** * [64]	2010	215	17–31	30	Students	236 apex images
**CK+** * [47]	2010	123	18–50	-	Three ethnicities (Euro-American, Afro-American and other)	593 sequences
***MMI-V*** * [65]	2010	25	20–32	25	Three ethnicities (European, South American, Asian)	1 h and 32 min of data. 392 segments
***AVLC*** * [67]	2010	24	Average ages were respectively 30, 28 and 29 years	25	eleven ethnicities (Belgium, France, Italy, UK, Greece, Turkey, Kazakhstan, India, Canada, USA and South Korea)	1000 spontaneousaughs and 27 actedaughs
**AvID** * [48]	2009	15	19–37	-	Native Slovenian speakers	Approximately one-hour video for each subject
**AVIC** [49]	2009	21	≤30 and ≥40	25	Two ethnicities (Asian and European)	No. episodes 324
**DD** [50]	2009	57	-	30	19% non-Caucasian	No. episodes 238
*VAM-faces* * [81]	2008	20	16–69 (70% ≤ 35)	25	One ethnicity (German)	1867 images (93.6 images per speaker on average)
*FreeTalk* * [82]	2008	4	-	60	Originating from different countries and each of them speaking a different nativeanguage (Finnish, French, Japanese, and English)	No. episodes 300
***IEMOCAP*** * [68]	2008	10	-	120	Actors (fluent English speakers)	Two hours of audiovisual data, including video, speech, motion capture of face, and text transcriptions
**SAL** * [51]	2008	4	-	-	-	30 min sessions for each user
**HUMAINE** * [52]	2007	Multiple	-	-	-	50 ‘clips’ from naturalistic and induced data
**EmoTABOO** * [53]	2007	-	-	-	French dataset	10 clips
***AMI*** [69]	2006	-	-	25	-	A multi-modal data set consisting of 100 h of meeting recordings
**ENTERFACE** * [54]	2006	16	average age 25	-	-	-
		5	22–38			
		16	average age 25			
**RU-FACS** [56]	2005	100	18–30	24	Two ethnicities (African-American and Asian or Latino)	400–800 min dataset
***MMI*** * [66]	2005	19	19–62	24	Three ethnicities (European, Asian, or South American)	Subjects portrayed 79 series of facial expressions. Image sequence of frontal and side view are captured. 740 static images/848 videos
**UT-Dallas** * [55]	2005	284	18–25	29.97	One ethnicity (Caucasians)	1540 standardized clips
**MIT** [57]	2005	17	-	-	-	Over 25,000 frames were scored
*EmoTV* * [83]	2005	48	-	-	French	51 video clips
**UA-UIUC** * [58]	2004	28	Students	-	Students	One video clip for each subject
**AAI** [59]	2004	60	18–30	-	Two ethnicities (European American and Chinese American)	One audiovisual for each subject
**Smile dataset** [60]	2001	95	-	30	-	195 spontaneous smiles

**Table 6:** Micro-expressions datasets. Number subjects: Number of subjects. Ages: age range of the subjects. FPS: frames captured per second. -: No Information. Samples: micro-expressions. Content: *Spontaneous* or *in-the-wild*.

Dataset	Year	Number Subjects	Age	FPS	Ethnicity	# of Data/Frames	FACs Coded	Samples	Lights	Resolution	Emotions
**SAMM** [92]	2018	32	average 33.24	200	Thirteen ethnicities (white British and other)	338 micro movements	Yes	159	Twoights as array of LEDs	2040 ×1088	Seven emotions. Macro/Micro
**CAS(ME)2** [93]	2018	22	Average 22.59	30	One ethnicity	250 macro, 53 micro	No	53	Twoight-emitting diose (LDE)ights	640×480	Four emotions. Macro/Micro
*MEVIEW* [84]	2017	16	-	25	-	31 videos	Yes	31	-	-	Five emotions. Macro/Micro
**Silesian deception** [94]	2015	101	Students	100	Third and fourth year students	101 videos 1.1 M frames.	Yes	183 micro-tensions	Proper illumination	640×480	Macro/Micro
**CASME II** [95]	2014	26	Average 22.03	200	One ethnicity	Among 3000 facial movements	Yes	247	Four selected LEDamps under umbrella reflectors	640×480	Five emotions
**CASME** [86]	2013	35 (19 valid)	Average 22.03	60	One ethnicity	More than 1500 elicited facial movements	Yes	195 in Class A, 100 in Class B, 95	Class A: naturalight, Class B: room with two LEDights	Class A: 1280×720. Class B: 640×480	Seven emotions
**SMIC-E: HS VIS NIR** [96]	2013	HS: 16	(22–34)	100	Three ethnicities (Asians, Caucasians and African)	Longest micro-expression clips: 50 frames	No	164	4ights at the four upper corners of the room	640×480	3 emotions (positive, negative and surprise)
			VIS: 8	25		Theongest micro-expression clips: 13 frames			71		
			NIR: 8	25		Same as VIS			71		
**SMIC** [97]	2011	6	-	100	-	1,260,000 frames	No	77	Indoor bunker environment resembling an interrogation room	640×480	Five emotions: Micro
**Canal9** [98]	2009	190	-	-	-	70 debates for a total of 43 h and 10 min of material	-	24	-	720×576	Political debates recorded by the Canal 9ocal Switzerland TV station
**YorkDDT** [99]	2009	9	-	25	-	20 videos for a deception detection test (DDT). seven frames	No	18	-	320 × 240	Two emotion classes
